# The invention of the psychosocial: An introduction

**DOI:** 10.1177/0952695112471658

**Published:** 2012-12

**Authors:** Rhodri Hayward

**Affiliations:** Queen Mary, University of London, UK

**Keywords:** Psychosocial, social psychology, psychoanalysis, planning, Suttie, Huxley, Halliday

## Abstract

Although the compound adjective ‘psychosocial’ was first used by academic psychologists in the 1890s, it was only in the interwar period that psychiatrists, psychologists and social workers began to develop detailed models of the psychosocial domain. These models marked a significant departure from earlier ideas of the relationship between society and human nature. Whereas Freudians and Darwinians had described an antagonistic relationship between biological instincts and social forces, interwar authors insisted that individual personality was made possible through collective organization. This argument was advanced by dissenting psychoanalysts such as Ian Suttie and Karen Horney; biologists including Julian Huxley and Hans Selye; philosophers (e.g. Olaf Stapledon), anthropologists (e.g. Margaret Mead) and physicians (e.g John Ryle and James Halliday).

This introduction and the essays that follow sketch out the emergence of the psycho-social by examining the methods, tools and concepts through which it was articulated. New statistical technologies and physiological theories allowed individual pathology to be read as an index of broader social problems and placed medical expertise at the centre of new political programmes. In these arguments the intangible structure of social relationships was made visible and provided a template for the development of healthy and effective forms of social organization. By examining the range of techniques deployed in the construction of the psychosocial (from surveys of civilian neurosis, techniques of family observation through to animal models of psychotic breakdown) a critical genealogy of the biopolitical basis of modern society is developed.

In 1950, Henry V. Dicks, the deputy director of London’s Tavistock Clinic, announced that ‘mental health’ had become the goal of modern policy (Dicks, 1950: 3-4). Just as the values of freedom and democracy had animated previous generations of activists and statesmen, the ideal of psychological well-being, he argued, now provided the basis of a new political programme. Although Dicks’s vision was ambitious, it was not unique. Similar statements could be found in the writings of a broad section of politicians and scientists (Nuttall, 2003, 2006; Thomson, 2006). The faith in political planning that animated British progressives during the 1930s and 1940s was matched by a concomitant belief that human nature itself could be improved through a coordinated series of psychological interventions (Marwick, 1964; Ritschel, 1997; Hayward, 2012). War-time social experiments in workplace democracy, personnel selection and infant welfare were predicated upon this belief that human potential could best be realised through central political direction and control (Ahrenfeldt, 1958; Rose, 1990). This conflation of the parallel projects of social and psychological reconstruction was made possible by the ill defined and over-determined concept of the ‘psychosocial'. It is this category, along with the auxiliary concept of ‘stress’, that is explored in this special issue of *History of the Human Sciences*. 

## The changing meaning of the psychosocial

The compound noun, ‘psychosocial’, first emerges in the 1890s — although a metaphysical insistence on a deep and mutually constitutive connection between personality and social life had been a commonplace throughout the nineteenth century. Victorian moralists drew from the philosophies of Plato and Hegel an organic vision of society that emphasised the close involvement of self and community (Harris, 1992; Collini, 1990). This vision was upheld by a broad swathe of cultural commentators, from radical socialists through to Liberal Anglicans, who agreed that working practices and social relationships were constitutive of human personality (Burkitt, 1991: ch. 1; Hayward, 2007: ch. 1). Although they differed over the precise nature of this process of constitution, these proponents held up the experience of fellowship with friends, family or nation as the essential and defining aspect of human kind, whereas independence and alienation became the markers of psychological ill health (Douglas-Fairhust, 2002: ch. 3; Clark, 1988).

The emergence of psychology and sociology as academic disciplines at the end of the nineteenth century challenged such moral understandings of personality. There had been attempts in the work of Moritz Lazarus and Wilhelm Wundt to launch a form of folk psychology which could provide objective representations of this communal experience; however, the gradual institutionalisation of both psychology and sociology ended such experiments (Klautke, 2010; Lepenies, 1988). In their stead the pioneers of both disciplines engaged in a period of intensive boundary work (Cavalletto, 2007: ch.1). Where Weber and Durkheim would rule out reference to psychological forces in the explanation of social phenomena, early psychologists, including Freud, attempted to depict social institutions as the outcome of emotional conflicts or developmental processes. Despite this territorial struggle, a faith in the close relationship of the sociological and the psychological persisted and by the early 1890s, the relationship took on a new substance and connotation. The statistical techniques that became the central research tools in both disciplines provided new ways of imagining and representing the psychosocial (Bulmer *et al.*, 1991; Danziger, 1990; Desrosières, 1998: ch. 5)

In early descriptions, the word ‘psychosocial’ had two connotations. In the writings of ethologists and criminologists, it described a complex of factors which exist on the boundary of psychology and sociology, such as religious rituals and sexual mores (McLane Hamilton, 1900: 550; Gumplowicz, 1999[1899]: pt. 2, ch. 1). On a second, more complex level, in the writings of the writings of early psychologists such as J. M Baldwin and G. Stanley Hall, the term was used to describe the developmental stage around adolescence in which childish individualism is replaced by a sense of communal duty and integration: part of that moment in adolescence when the individual entered into the life of the race (Baldwin, 1895; Hall, 1905: II, 342-54). Such approaches, in the hands of later psychological commentators such as Freud and William McDougall, left social life dependent upon the play of biological instincts. 

Left wing psychoanalysts led the resistance to the prioritising of the biological over the sociological in the explanation of individual and social behaviour. In the United States, Trigant Burrow, Karen Horney and Frankwood Williams used psychodynamic theories to sustain a radical critique of contemporary forms of social organisation (Burrow, 1927; Horney, 1927; Williams, 1934). In a move that would anticipate the arguments of the Frankfurt School, they sought to demonstrate the psychopathological consequences of modern capitalism while demonstrating the foundational role of social life in the constitution of the psyche. In Britain, these arguments received their clearest articulation in the work of the heterodox Glaswegian psychoanalyst, Ian Suttie. Indeed, it is Suttie who established the term in British psychological discourse (Suttie, 1922). A committed socialist and feminist, Suttie was unhappy with the orthodox psychoanalytic position characterised by Freud’s attempt to explain group solidarity through reference to the reproductive instinct. This was evident, Suttie believed, in the origin myths propounded in *Totem and Taboo *(Freud (1990[1913])), in which Freud described the social group emerging from the shared sexual jealousy and fear exhibited by a ‘young ‘band of brothers’ toward their primal father. Suttie sought to displace this Oedipal narrative, rooted in a patriarchal framework, with a matriarchal theory which explained the growth of social bonds as an extension of the child’s love for his or her mother (Suttie, 1960[1935]).

Freud’s error, Suttie believed, was his uncritical adoption of the evolutionary theory of mental and racial recapitulation advanced by the German embryologist, Ernst Haeckel. Writing in the 1860s, Haeckel had claimed that the ontogenetic development of the individual recapitulated the phylogenetic development of the race. The infant shared in the primitive and magical thinking of uncivilised races whereas adults strove toward the enlightened rationality of modern Europeans. Suttie, by contrast, argued that forms of social organisation were not predetermined by the prehistory of the race but were in fact accidents of culture (Suttie, 1924). There was, he thought, nothing natural or inevitable about patriarchy or the Oedipus complex: these were in fact distortions peculiar to Western civilisation. He looked to anthropological studies of the Arunta people of Australia to demonstrate the possibility of alternative forms of social and emotional organisation. Belief in the possibility of matriarchy received further succour from Margaret Mead's studies of Western Samoa (1928) and New Guinea (1935), and Robert Briffault’s wide ranging anthropological study, *The Mothers* (1936) (Passerini, 1999: ch. 4). Such studies underlined the idea that social organisation and psychological development were not predetermined by biological inheritance but contingent upon a series of historical factors. As Suttie made clear, the psychological and the sociological could not be disentangled. He argued in *The Origins of Love and Hate* that ‘psychologists are prone to describe mind as if it were an independent, self-contained but standardised entity, a number of which, grouped together in some mysterious way, constitutes a Society. The separation of the science of the Mind from that of Society is arbitrary’ (Suttie, 1960[1935]: 12-13). 

This conflation of Mind and Society had two implications. First, it extended the possibility of therapy. Where Freud had insisted that the neurotic individual should be cured through the interrogation and recovery of their personal history, social psychologists and anthropologists insisted that individual personality could be remade through the creation of new forms of social organisation (Thomson, 2006: ch. 7; Overy, 2009: ch. 4). Drawing on the vocabulary of contemporary neurology (which itself was caught up in the language of British idealism), it became commonplace in interwar commentaries to hold up social integration as the key to psychological health (Stapledon, 1939: 292-99; Eliot, 1956; Smith, 2003). Second, following on from this, the conflation of mind and society promoted an implicit hierarchy in which the social took priority over the biological (Smith, 2003). Escaping the claims of Haeckelian recapitulation that had held Freud in thrall, Suttie, Burrow and others were able to argue that cultural development had superseded biological evolution. As Suttie noted: ‘… it is glaringly obvious that the elaboration and accumulation of tradition and the devising of new ways of training and applying thought are quite independent of cerebral evolution’ (1924: 144). This dual understanding of the psychosocial would assume a central place in the philosophies of the two major British writers on the concept in the 1940s: James Halliday and Julian Huxley. 

## Halliday and Huxley: The psychosocial and the limits of biology

From the late 1930s until the 1950s, James Halliday commanded a broad audience in his attempt to the marry psychosomatic diagnoses advanced by Flanders Dunbar and Franz Alexander with the new epidemiologically grounded social medicine promoted by radical physicians and political reformers (Porter, 1996). Working as an National Health Insurance investigator for the Glasgow Regional Health Board, Halliday saw the changing distribution of sickness claims recorded in the Department of Health’s annual returns as an index of the population’s declining psychological state. The growing level of psychopathology made manifest in the insurance returns could only be countered, Halliday believed, through a wholesale social and political reformation (Hayward, 2009). In Halliday’s writings, as Andrew Hull makes clear in his contribution to this issue, the psychosocial is described as a kind of miasma of historical experience in which accumulated episodes of frustration and disappointment encourage the individual’s flight into illness. His analysis went beyond the individualistic models of primary and secondary gain developed in Freudian theory. Rather he depicted such psychopathological reversals as environmental responses experienced across large sections of the population (Halliday, 1949; Galdston, 1954).

The idea of the psychosocial as a new form of environment was taken up by the British biologist, Julian Huxley. Huxley shared Suttie’s conviction that the human species had escaped biological determinism, believing instead that it had entered an accelerated state of ‘psychosocial evolution’ characterised by the ever increasing frequency of cultural change (Huxley, 1942). This hierarchical vision of biological, psychological, social and moral levels of reality had been present in Huxley’s writings from his early work on *The Individual and the Animal Kingdom* (1912), but by the Second World War, his concept of the psychosocial as a higher level of evolutionary development had taken on a more definitive form. Drawing on the ideas of the popular science broadcaster Gerald Heard, he argued that human evolution would involve the subjugation of man’s animal impulses to his conscious needs (Heard, 1939). It demanded the surrender of individual aims to group ideals (Huxley, 1941). Evolutionary progress had moved from the level of the biological to the cultural and its realisation was dependent upon foresight, planning and control (Huxley, 1942: 571-78; 1966[1964]: 76). Although Huxley remained cautious about the over extension of biological analogies, as Mark Jackson shows in his contribution to this collection, it is striking how closely the next imagined stage in evolution matched the programmes of political reform sketched out by his Labour and Fabian contemporaries and his own colleagues in Political and Economic Planning (P.E.P. (Marwick, 1964)). It involved achievement of a higher form of personality through the rational control of biological resources.

## The historiography of the psychosocial

In tracing the emergence of this new conceptual apparatus and the forms of identity that it made possible, the essays included here build upon the extensive historical inquiries carried out by Nikolas Rose and other members of History of the Present Network. Inspired by the work of Jacques Donzelot and Michel Foucault, these writers provided provided rich (and sometimes dazzling) accounts of the role of psychological knowledge in the constitution of modern welfare states. They concentrated on the way that psychological and sociological language and techniques made available new forms of identity and new kinds of social relationship. The writings of Rose and Peter Miller followed Foucault and Gilles Deleuze in emphasizing the productive effects of power (Rose, 1990; Miller and Rose, 2008). In their accounts, the rise of the psychological sciences made tangible a new domain of government populated with novel objects and forces.

More recently, revisionist social historians (notably Joanna Bourke and Mathew Thomson) have contested the defined historical narrative put forward by Rose and others. Instead of focusing on the relationship between subjectivity and government, these authors have sought to emphasise the multiplicity of actors and the various agendas involved in the production of psychological knowledge (Bourke, 1996; Thomson, 2006, 2007). Whereas Rose and Miller emphasize the role of state agencies such as Child Guidance Clinics or military selection boards in the production of a new psychological framework, Thomson and Bourke stress the parts taken by autonomous groups, play and popular entertainment in creating spaces in which new identities can be articulated and new transformative techniques explored (Thomson, 2006, 2007). These spaces, as Bourke has argued, make possible new forms of embodied experience which in turn provide the grounds for new psychological categories (Bourke, 2003, 2005).

## Imagining the psychosocial

While the articles in this special issue all draw upon Foucauldian and revisionist approaches, their emphasis is different. Indebted to recent work in the history of science and medicine, their authors pay closer attention to the particular local conditions that give rise to new theories of psychosocial reconstruction, and the tools and concepts that have made such theories possible. They address four major questions: How is the psychosocial imagined? What tools are involved in making it visible? What values are encoded in the concept and how is the relationship between the category of the psychosocial and the biological imagined?

Of the contributions to this issue, two focus upon North American developments and the other five upon the United Kingdom. Mark Jackson opens the collection with a study of how Hans Selye, the champion of stress theory, extended his physiological studies to rationalise utopian schemes of social re-organisation, while Ed Ramsden explores the U.S. ethologist, John B. Calhoun’s attempt to model new forms of urban development in laboratory rat experiments. In the British studies, Andrew Hull examines James Halliday’s work, arguing for its location in a particular Glaswegian tradition of medical holism. Edgar Jones and Ian Burney both focus upon wartime investigations into the effects of aerial bombardment while Jonathan Toms and Teri Chettiar consider the role of the psychosocial in postwar mental hygiene and family therapy. 

All these articles emphasise how the disruption wrought by war and modernity rendered the psychosocial visible. Slum clearance, aerial bombardment or organised labour schemes, as Ramsden, Hull and Burney show, transformed the social environment into a kind of experimental laboratory in which the effects of different test conditions upon a population could be measured and compared (c.f. McGonigle and Kirby, 1936). Moreover, it was the administrative machinery of the welfare state, developed (arguably) around the problems of modernity, that produced the vast aggregations of personal data, in doctors' records and insurance returns, that served as indices of the nation’s psychological health. These new forms of administration, as Edgar Jones makes clear, did not simply record the data upon which psychosocial theories would be grafted: they instead produced it. Wartime investigations into civilian and military neuroses elicited new patterns of somatisation -- gastric disorder in particular. As many of these studies show, the space of psychosocial analysis was opened up by the bodies and behaviour of men, women and animals under investigation.

The vast range of symptoms and behaviours recorded in the administrative machinery of the welfare state were rendered meaningful through the concept of stress. Whereas Freudian models of the unconscious have provided an imaginative mechanism for joining personal characteristics and physical disturbances to episodes in the individual past, stress escaped the individual frame. It provided a kind of conceptual glue which allowed individual failings -- whether physical, mental, social or intellectual -- to be joined to broader transformations in society or the environment. Stress is thus a productive concept allowing any number of experiences, institutions and events to be joined together to create a new landscape of meaning.

## The values of the psychosocial

As seen in our discussion of Huxley and Halliday, a hierarchical vision of the relationship between culture and biology was implicit in the concept of the psychosocial. The concept also encoded a normative model of human relationships. This model, as Toms and Chettiar make clear, drew heavily on mid twentieth-century ideas of the family. Family provided a useful resource for connecting emotion and power, for setting out criteria for development and for thinking through the pathological or enabling effects of dependence and independence. Family had come under close psychiatric scrutiny during the war, as the evacuation of children and the separation of couples through mobilisation constituted a kind of natural experiment. In Bowlby’s work on evacuees, Woodside and Slater’s studies of separated families and Curle and Trist’s work on the domestic disruption created by returning prisoners of war, the family structure was revealed as delicately balanced between competing psychological needs (Savage, 2011). Although the idea of psychosocial health was predicated upon the idea of the family, it was, as Chettiar demonstrates, a historically specific idea rooted in twentieth-century notions of the companionate marriage and of equality (c.f. Langhammer, 2007). 

## The psychosocial and the return of biology

In the writings of Suttie, Huxley and others, the concept of the psychosocial had been used to limit the claims of biology. It allowed for a vision of human progress to be developed which overrode ideas of racial evolution and claims of the superiority of particular ethnic groups sustained by these ideas. By the 1960s, however, this attempt to use the psychosocial to limit the jurisdiction of biology had all but failed. As Mark Jackson shows in his essay on Selye, the languages of biology and sociology remained closely bound, as scientists and social commentators turned to examples from physiology and cell biology to imagine and describe complex social processes. And it was this same metaphorical co-dependence that opened up once again the possibility of re-describing social relationships in biological terms. By the early 1960s, ethologists and evolutionary psychologists were articulating a new understanding of biological politics that would in the decades that followed eclipse the claims of the psychosocial (c.f. Harraway, 1990; Segerstrale, 2001). This vision of biology was itself very different from the version that had developed in the interwar period. It took the material of the psychosocial -- questions of rank, hierarchy, dependence and personal distress -- and recast it in biological terms. And through this process, it made available a new set of signs and objects through which society, health and human relationships could be imagined. The return to biological objects (such as cortisol counts or FMRI scans) to measure social relationships in some ways marks the end of the psychosocial project. Certainly contemporary schemes of psychological welfare, such as those developed around the happiness agenda and wellbeing economics championed in Western democracies, increasingly rely upon biological models of emotion and mental disorder to ground their programmes. A concept which had been used to mark the limits of biological explanation in the years after the Second World War is now itself imagined in biological terms. 
